# Predicting protein–ligand binding affinity and correcting crystal structures with quantum mechanical calculations: lactate dehydrogenase A†
†Electronic supplementary information (ESI) available: *xyz* coordinates for theoceptor complexes, and additional information as noted in the text. See DOI: 10.1039/c8sc04564j


**DOI:** 10.1039/c8sc04564j

**Published:** 2019-01-04

**Authors:** Iva Lukac, Hend Abdelhakim, Richard A. Ward, Stephen A. St-Gallay, Judith C. Madden, Andrew G. Leach

**Affiliations:** a School of Pharmacy and Biomolecular Sciences , Liverpool John Moores University , Byrom Street , Liverpool , L3 3AF , UK . Email: a.g.leach@ljmu.ac.uk; b Chemistry, Oncology, IMED Biotech Unit , AstraZeneca , Cambridge , UK; c Sygnature Discovery Ltd , Bio City, Pennyfoot St , Nottingham , NG1 1GF , UK

## Abstract

Accurately computing the geometry and energy of host–guest and protein–ligand interactions requires a physically accurate description of the forces in action. Quantum mechanics can provide this accuracy but the calculations can require a prohibitive quantity of computational resources. The size of the calculations can be reduced by including only the atoms of the receptor that are in close proximity to the ligand. We show that when combined with log *P* values for the ligand (which can be computed easily) this approach can significantly improve the agreement between computed and measured binding energies. When the approach is applied to lactate dehydrogenase A, it can make quantitative predictions about conformational, tautomeric and protonation state preferences as well as stereoselectivity and even identifies potential errors in structures deposited in the Protein Data Bank for this enzyme. By broadening the evidence base for these structures from only the diffraction data, more chemically realistic structures can be proposed.

## Abstract 2

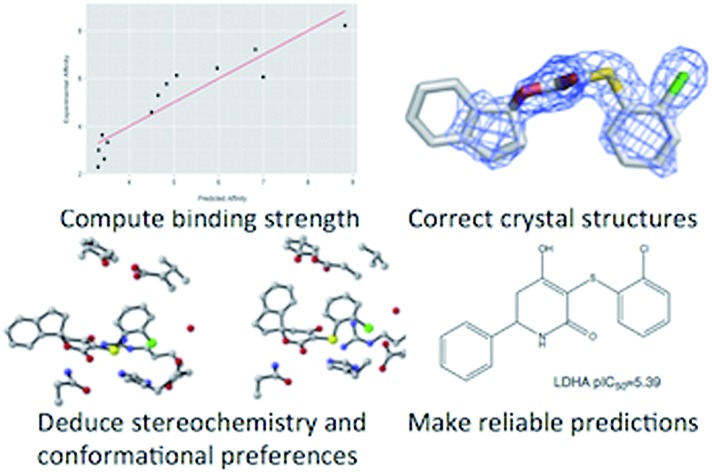
Quantum calculations plus lipophilicity (log *P*) lead to usefully accurate predictions of binding affinity that allow correction of crystal structures.

## 1 Background

The interaction of small guest molecules with larger hosts in aqueous systems is a key component of many applications including: sensors for *in vivo* use,^1^ enzyme catalysis,^2^ antibody recognition,^3^ sequestration,^4^ purification of waste streams,^5^ environmental remediation,^6^ drug design,^7^ toxicology,^8^ and supramolecular chemistry.^9^ The structures of such complexes are routinely studied with X-ray diffraction experiments and those involving proteins are deposited in the Protein Data Bank (PDB).^10^ These have been investigated to learn about how proteins and ligands interact.^11^ Such studies presume that the structures are correct and of high quality based on descriptive statistics describing how well the structure can explain the observed diffraction data. Ourselves and others have found that these statistics are not sufficient – chemically infeasible ligand structures can achieve apparently better statistics.^12^ Quantum mechanical (QM) calculations can provide a complementary guide to the chemical reasonableness of structures. A recent study illustrates the challenge: Kumar *et al.* contrasted quantum mechanical calculations with deposited structures and found a divergence between complexes of arginine (which behaved as predicted) and lysine (which did not); the contrasting likelihoods of these two residue types being clearly identifiable in the electron density was not considered but could provide an alternative interpretation of their findings.^13^ Routinely allowing quantum calculations to decide when to go beyond reliance on the diffraction data alone would increase the likelihood of identifying the correct structure but requires a method that can predict binding strength to a useful level of accuracy. Here we propose such a method and show how it has guided us to improved structural interpretations for an enzyme of pharmaceutical interest. This has permitted us to begin the process of deducing when differences in diagnostic statistics, such as *R*_work_ and *R*_free_ are not significant. Others have previously lamented the reliance on these statistics and particularly when they distract from achieving a sound chemical interpretation that correctly accounts for the physical interactions at play.^14^

Correctly quantifying the interaction strength between a host and a guest or a protein and a ligand computationally requires a physically accurate description of the molecules, of the kind provided by QM calculations. Despite significant progress, such calculations remain challenging because of their computational cost. Including only the portion of the host or protein directly involved in binding reduces this cost and examples of this approach are mentioned in recent reviews.^15^,^16^ Related calculations see the protein or ligands split into fragments that can then be treated with QM.^17^–^22^ Geometries generated by a molecular dynamics simulation can be treated with QM to compute affinities.^23^ Alternatively, much faster QM methods (which are usually less general or accurate) can be applied to larger portions of the system, or empirical corrections applied to improve accuracy with minimal increased computing cost.^24^–^26^

Alternative approaches to understanding the behavior of proteins and quantifying their interaction strength with ligands have been pursued vigorously over the past decade. The majority of this effort has been in the area of simulation and recent advances in free energy perturbation (FEP) are particularly noteworthy.^27^,^28^ One significant issue with these methods is that (unlike quantum approaches) the parameterization schemes that underpin the description of the ligands often require individual tailoring to the molecules being studied.^27^ Like quantum calculations, simulations require a significant amount of computational time to compute binding energies.

Structural biology studies often entail generating crystalline solids containing protein–ligand complexes. These are irradiated to create X-ray diffraction patterns, arising from interactions with electrons in the molecules. Software is employed to link from atomic coordinates to electron density and hence X-ray diffraction patterns; thus a likely set of atomic positions can be identified. These interpretations can be problematic and are rarely unambiguous.^12^ QM calculations can support these interpretations but it is usual for improved agreement between predicted and observed diffraction patterns to determine the structure deemed to be correct.^29^–^34^ One way to meld the chemical insight provided by quantum chemical calculations with the experimental data available from a diffraction experiment is to iteratively include quantum calculations in the refinement procedure; higher quality structures of proteins and ligands have been achieved in this way using the quantum refinement approaches pioneered by the Ryde group.^30^ This has proved particularly effective for metalloproteins and for assigning protonation states.^15^,^30^,^35^–^37^ Indeed, QM-derived ideas have been influencing structural refinement for nearly a century. Sometimes, the diffraction data cannot determine the correct structure and this is particularly the case for novel ligands whose behavior is much less well understood than that of proteins.^38^ One way alternative interpretations of the diffraction data can be assessed is by examining ligand omit maps that are created by using the coordinates of the protein to generate phases and to use these to determine the ligand electron density that is unaccounted for.^39^–^41^

We propose an approach in which a correlation with computed log *P* values provides a usefully accurate treatment that accounts for the differences between binding energies computed with quantum mechanics and experimentally observed values. We style the resulting type of calculation as a “theoceptor” (theoretical receptor) by analogy to the theozymes (theoretical enzymes) of Houk and co-workers.^42^,^43^ We show that the approach can be applied to predicting aqueous host–guest interaction energies in the realm of biological or supramolecular chemistry. We also present a theoceptor for the enzyme Lactate Dehydrogenase A (LDHA) and demonstrate the value that theoceptor calculations can provide in terms of detailed structural insights and improved crystallographic structures.

The current study is presented in three parts. In the first, we describe our approach. Subsequently, we describe our theoceptor for LDHA. Finally, we show how these calculations can increase the value of protein–ligand crystal structures and provide alternative interpretations of the electron density for several examples taken from LDHA.

## 2 Results and discussion

Quantum mechanical calculations generally deal with the gas phase electronic energy of the system whereas experimental observations in biological systems are related to free energies in solution (eqn (1)).

1 Δ*G*_bind_(aq) = Δ*E* + Δ*H*_corr_(gas) – *T*Δ*S*(gas) + ΔΔ*G*_solv_

The free energy of binding in solution (Δ*G*_bind_(aq)) is related to the change in gas phase electronic energy (Δ*E*, eqn (2)) computed with QM. Corrections (Δ*H*_corr_) are added to obtain gas phase enthalpies and a gas phase entropy change (Δ*S*(gas)) is computed. The change in solvation free energies (ΔΔ*G*_solv_) completes the link to the experimental environment.

2 Δ*E* = *E*_complex_ – *E*_ligand_ – *E*_receptor_

**Fig. 1:**
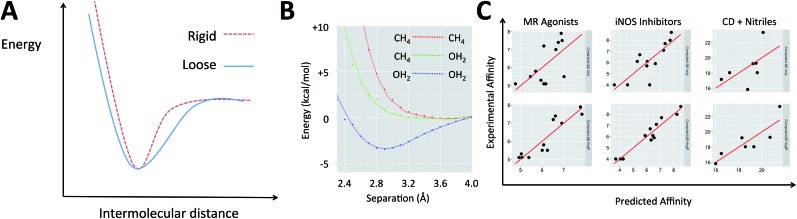
(A) The difference between a rigid and a loose complex illustrated by the change in energy as ligand and receptor approach one another. (B) Variation in electronic energy (M06/6-31+G**) as non-polar groups and/or polar groups approach one another. (C) Example protein–ligand and host–guest systems studied with QM calculations. Top: experimental affinity is plotted against computed energies, Δ*E*; bottom: log *P* term is added.

The electronic energies in eqn (2) can account for the energy of the complex but do not indicate the shape of the potential energy surface (PES), which describes how rigid the complex is. When two molecular species come into proximity, the potential energy surface can involve a narrow minimum or a wide one (Fig. 1A). A narrow minimum corresponds to a rigid complex and a greater entropy penalty than a wider minimum. Different interaction types make different contributions to the shape of the PES. Density functional theory calculations were used to investigate how the energy of the system changes as several functional groups approach one another. The full set of calculations is described in the ESI (section S1†) but the key results can be summarized by considering water and methane as examples of polar and non-polar functional groups, respectively (Fig. 1B). Only when two polar groups (appropriately oriented) or charged groups of opposite sign approach one another is there a well-defined energy minimum; only polar–polar interactions tend to increase complex rigidity. It is of course the case that even many polar groups will have a repulsive interaction when the polarities are mismatched and that two non-polar groups that are aromatic rings can give a shallow energy minimum.^44^ To understand the thermodynamics of a gas phase binding event (Δ*H*_corr_(gas) – *T*Δ*S*(gas)), it is important to consider the relative contribution of polar and non-polar groups. Furthermore, the solvation free energy of the ligand and of the complex in water will be enhanced by the presence of polar groups on the ligand and diminished by non-polar regions. The solvation free energy of the protein is a constant and so accounting for whether the groups involved in binding are polar or non-polar offers a simple way to account for the change in solvation free energy (ΔΔ*G*_solv_).

Medicinal chemists have been concerned about the influence of non-polar groups upon binding affinity for some time and log *P* has been used to differentiate ligands that rely primarily upon polar interactions from those that are hydrophobic.^45^–^47^ The partition coefficient, *P*, and its logarithm are convenient parameters because they are easily measured and can be accurately predicted.^45^ It is our hypothesis that a ligand's log *P* can serve as a surrogate for the three terms on the right of eqn (1) when computing its binding free energy.^48^ This leads us to propose that for a given system, binding affinity can be described by eqn (3).

3 Affinity = *α*Δ*E* + *β* log *P* + *γ*

where *α*, *β* and *γ* are constants for that system. We propose obtaining values for these empirically and Δ*E* is defined by eqn (2), where *E* indicates a quantum mechanical energy that is obtained with no vibrational corrections included. The energy of the receptor (*E*_receptor_) in this equation is arbitrary because it is constant for all ligands but it will influence the resulting value of both *α* and *γ*. A range of affinity assessment types (IC_50_, *K*_i_, *K*_d_, *etc.*) can be used but should all be transformed such that they will have a linear relationship with energy. We do this by transformation to pIC_50_, p*K*_i_, p*K*_d_ such that improved affinity corresponds to increasing values of each of these properties; *α* should therefore be negative and *β* positive if more negative values of Δ*E* and increased lipophilicity correspond to tighter binding. In order to obtain values for the three constants, at least three compounds of known affinity are required and thus, like the linear interaction energy approach, only relative affinity can be computed.^49^ Δ*E* would usually be computed using the lowest energy protein–ligand complex and lowest energy conformation of the free ligand and therefore, be relevant to cases where the bound state is dominated by one geometry.^16^ However, the approach can be simply adapted to include multiple low energy conformations (of bound or free states) using a Boltzmann weighting scheme. The flexible form of eqn (3) naturally ameliorates other deficiencies of the calculations including the exclusion of longer range interactions (primarily electrostatic) with the regions of the protein that are not included in the theoceptor and deficiencies in the continuum part of any solvation models that are used (such as inappropriate values of the dielectric of the medium). It is challenging to predict the dielectric that the protein environment provides and so we take advantage of this approach by treating the protein using gas phase calculations with no continuum solvation.

The effect of eqn (3) is that for a polar ligand that makes a full set of polar interactions with the receptor, Δ*E* will be large and negative and this will offset the low log *P* term. A polar ligand that does not make a satisfactory set of polar interactions will have a less beneficial Δ*E* term that will not be sufficient to counteract the small log *P* contribution. A largely hydrophobic ligand will also have a smaller contribution to binding affinity from Δ*E* but this might be offset by the hydrophobicity term. There have been a number of reports in which quantum mechanical calculations have been used to compute binding energies for protein–ligand and other host–guest interactions. Energy differences are computed with no vibrational corrections and provide data with which this concept can be tested (in certain cases, the log *P* values have been computed by ourselves). The examples (Table 1) include: (A) Roos *et al.* who looked at the relative binding affinities of a matched series of mineralocorticoid receptor (MR) agonists, using a range of DFT methods, with M062X performing the best.^50^ (B) Investigation of inhibitors of the dimerisation of inducible NO-synthase by Leach *et al.*^51^ This system includes backbone atoms of six residues forming the binding site, an iron porphyrin and its attached cysteine thiol. Among the QM methods tried, M06HF performed the best. (C) Roos *et al.* who used DFT to explore the relationship between the measured and predicted affinities for a set of positively charged amidine and guanidine cores binding to the β-site of APP cleaving enzyme (BACE-1).^52^ (D) Hylsová and co-workers, who have studied a series of pyrazolopyrimidine inhibitors of the kinase CDK2.^53^

**Table 1:** Protein–ligand and host–guest systems (see text) studied by QM and reevaluated by ourselves. Pearson's correlation coefficients (*R*^2^) and root-mean-squared errors (RMSE) describe the link between affinity and Δ*E* when combined with log *P* or a random number. The coefficients for eqn (3) are also provided

System	Δ*E* + random		Δ*E* + log *P*		*α*	*β*	*γ*
	*R* ^2^	RMSE	*R* ^2^	RMSE			
A	0.51	0.85	0.83	0.51	–0.35 ± 0.01	1.58 ± 0.10	4.71 ± 0.17
B	0.67	1.00	0.93	0.46	–0.28 ± 0.01	1.22 ± 0.04	–6.68 ± 0.23
C	0.67	0.52	0.71	0.48	–0.32 ± 0.01	0.24 ± 0.01	–0.13 ± 0.01
D	0.61	0.65	0.82	0.45	–0.10 ± 0.00	0.88 ± 0.04	–9.50 ± 0.28
E	0.55	3.11	0.73	2.41	–0.08 ± 0.02	5.10 ± 0.78	–3.02 ± 1.84
F	0.33	2.37	0.75	1.45	–0.50 ± 0.16	3.39 ± 0.58	10.26 ± 1.35

As shown in Fig. 1C and section S2,† in each of these examples, incorporating log *P* improved the description of measured binding affinity by the QM energies. To make the comparison in the most straightforward way, two-parameter linear regression has been performed using firstly Δ*E* and calculated log *P* as independent variables and secondly Δ*E* and a random value that spans the same range as the log *P* values. The statistics for the second cannot be worse than those for the single parameter model using only Δ*E*. The resulting *R*^2^ and RMSE values are shown in Table 1. In all cases, the addition of log *P* improved the correlation. For system A, Roos *et al.* also performed FEP calculations and achieved a correlation with *R*^2^ of 0.60 and RMSE of 0.73 (worse correlation is found when correlating with p*K*_i_–log *D* instead of just p*K*_i_).^52^

The values obtained for the coefficients in eqn (3) are shown in Table 1, along with the standard deviation obtained from systematic leave-one-out analysis. It is likely not a coincidence that the BACE inhibitors (*β* = 0.24) are binding to a highly polarized dianionic binding site while the iNOS inhibitors (*β* = 1.22) bind to a site dominated by the hydrophobic porphyrin of heme and MR agonists (*β* = 1.58) are binding to sites which lack substantial polarity (a lone threonine provides a point of polar interaction). The CDK2 binding site (*β* = 0.88) includes hydrophobic residues and several polar interactions. The more hydrophobic the binding site, the higher the value of *β*.^48^ The same approach is also applicable to organic host–guest systems in water. For instance: (E) in their computational studies of a set of carboxylic acids binding to an octa-acid cavitand, Mikulskis *et al.*^54^ compute binding energies using several DFT methods and achieve their best results (before the addition of vibrational corrections) with BP86/TZVP when Cosmo solvation is included in their calculations. (F) Kimura, Yukiyama and Fujisawa studied the complexation of a series of nitriles by an α-cyclodextrin host in water and performed calculations at the B3LYP/6-31++G** level to rationalize their observations. In both cases, the agreement between computation and experiment is enhanced when eqn (3) is applied. In the case of these two organic hosts, the values of *β* are higher than for the protein–ligand systems. This likely reflects the absence of polar groups inside the cavities (unlike proteins which must contain amide groups). The sets of compounds studied in host–guest interactions are smaller than for the protein–ligand binding examples and this likely explains the larger standard deviations observed for the values of the coefficients in Table 1. Naturally, the organic hosts are more challenging to study with force field-based approaches because they would require parameters to be developed for both the host and guest.

**Fig. 2:**
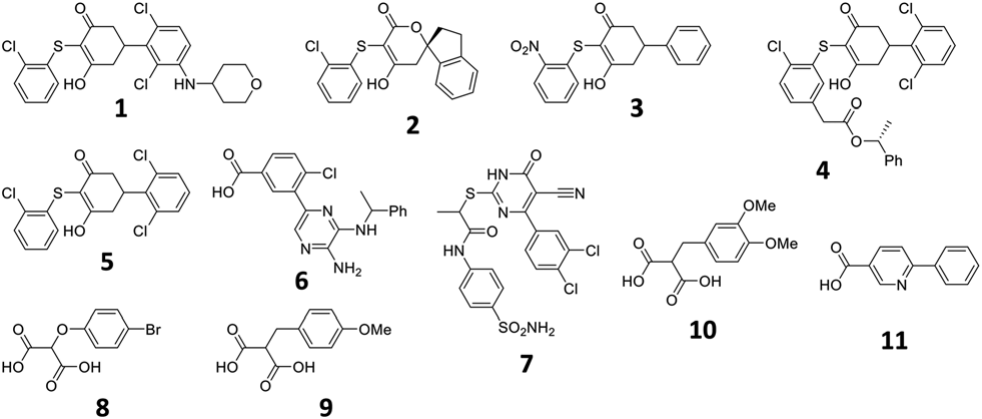
Structures described in the text.

To investigate the utility of the theoceptor approach, it has been applied to a system of therapeutic interest: Lactate Dehydrogenase A (LDHA). LDHA catalyses the reversible conversion of pyruvate to lactate, with the concomitant conversion of NADH to NAD+. Several classes of cancers are characterised by elevated levels of lactate, and LDHA is overexpressed in human tumors.^56^–^61^ As such, inhibitors of this enzyme have been sought as potential therapeutics. Several classes of inhibitors of LDHA have been described and a wealth of structural information and binding affinity data are available.^62^–^77^ The set of compounds (**1–11**) studied include seven from high-throughput screening (HTS) of the Genentech/Roche corporate compound collection;^66^,^68^,^69^,^71^,^72^,^78^ two small, negatively charged compounds;^73^ and three compounds sharing malonate as a common substructure originating from AstraZeneca's fragment screening approach (Table 2, Fig. 2).^74^ The selective and potent LDHA inhibitors discovered so far exhibit a common structural feature: a carboxylic acid moiety (or other acidic functional group) that is ionized at physiological pH. Structural studies reveal that in the binding site, this is placed in close proximity to where the acid in the substrates and products binds. All interact with the basic side-chain of Arg168.

**Table 2:** Compounds selected for computational studies with experimental values of pIC_50_ and p*K*_d_, PDB identifier and the chain label used to compute the RSCC value for each ligand. The clog *P* was calculated using the ChemAxon^55^ log *P* predictor and Δ*E* was calculated with the theoceptor method

Compound	pIC_50_	p*K*_d_	PDB code (chain)	RSCC	clog *P*	Δ*E* (kcal mol^–1^)
1	7.22	NC	4R69 (D)	0.943	5.58	–17.7
2	6.44	NC	4RLS (D)	0.883	4.97	–13.8
3	5.76	5.46^*a*^	4QO7 (A)	0.958	2.86	–23.2
4	8.22	NC	4R68 (B)	0.979	8.07	–15.0
5	6.06	5.74^*a*^	4QO8 (A)	0.921	6.15	13.6
6	5.3	5.3^*a*^	4M49 (A)	0.816	3.81	–10.3
7	6.12	5.29^*a*^	4JNK (D)	0.911	2.96	–24.9
8	<3.3	3.67^*a*^, 3.33^*b*^	4AJE (B)	0.988	2.06	–15.6
9	<3.3	2.96^*a*^, 3.63^*b*^			1.63	–18.9
10	<3.3	3.55^*a*^, 3^*b*^	4AJI (B)	0.963	1.47	–19.7
11	<2.7	2.63^*a*^	4I8X (B)	0.913	2.45	–10.4

^*a*^Obtained by SPR.

^*b*^Obtained by NMR

Structures of **1–7** are all available in complex with human LDHA.^66^,^68^–^70^,^72^ Crystal structures of **8** and **10** are available in complex with rat LDHA (PDB codes ; 4AJE and ; 4AJI),^79^ and the structure of **11** is available in complex with rabbit LDHA (PDB code ; 4I8X).^80^ It should be recalled that these structures are proposed interpretations of the observed electron density and alternative interpretations may also be reasonable. While global metrics such as resolution have their place for assessing the structures, more localised values prove instructive when focusing on the ligands. One of the most common metrics for assessing the fit of a proposed structure to the local electron density is the real space-correlation coefficient (RSCC). It ranges from 0 (‘bad’, electron density is effectively missing) to 1 (‘good’, model fits the density perfectly).^81^–^83^ RSCC values for the selected ligands are given in Table 2. Density maps contoured at 1*σ* of the ligands with lower RSCC values (ligands **1**, **2**, **6** and **7**) are shown in section S5.†

**Fig. 3:**
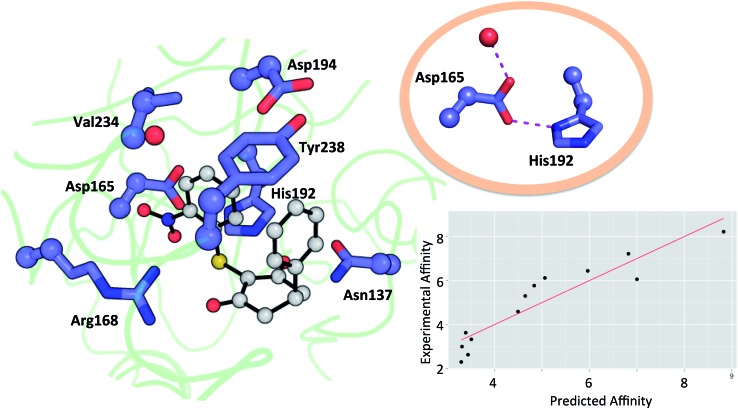
Theoceptor for the LDHA nicotinamide site taken from the complex with **3** (shown in grey). C_α_, C_β_ (spheres) and water oxygen (red sphere) were fixed during the QM optimization. The insert shows the hydrogen bonding network involving a water molecule, Asp165 and His192. Measured affinity is plotted against computed affinity for compounds **1–11**, oxamate and malonate. *R*^2^ = 0.85 and RMSE = 0.76.

Residues directly involved in ligand binding were selected as a part of the theoceptor: Arg168, His192, Val234, Asp165, Asp194, Tyr238, and Asn137 (Fig. 3). These were extracted from the structure deposited in the PDB with the code ; 4QO7 (theoceptors derived from other protein structures performed equivalently). Protonation states were assigned such that Arg168 is protonated, Asp165 and Asp194 are both deprotonated and His192 is protonated in order to form a stabilizing interaction with Asp165 and the net charge of the residues was 0. There are two noteworthy changes in the sidechain conformations amongst the deposited structures but one arrangement is clearly most relevant to the bound state in humans: Arg105 protrudes into the binding site in rat and rabbit structures as well as in the apo form of human LDHA but in the holo forms of the human protein, it interacts with Glu191 instead (and so it is excluded from the theoceptor) while Tyr238 moves out of the binding site only in the apo form (and hence is included). Asp165 forms a hydrogen bond with His192 and is stabilised by an additional hydrogen bond with one water molecule. Preliminary studies showed that the water molecule maintains the sidechain in an optimal position to interact with the His192 sidechain and so is considered structural and was kept in the theoceptor (Fig. 3, insert). All interactions with the ligand are through protein sidechains so backbone, cofactor and the remainder of the protein were deleted. To account for the scaffolding effect of the rest of the protein, C_α_, C_β_ and water oxygen atoms were fixed in space using the fixed Cartesian redundant coordinate feature in Gaussian 09,^84^ as shown in Fig. 3. The geometries were optimized *in vacuo* at the M06 level of theory with the 6-31+G** basis set because this level of theory should provide a well-balanced treatment of different interaction types.^85^,^86^ Free ligand structures were optimized with solvation incorporated, using the PCM water model, at the same level of theory. Δ*E* values were defined as in eqn (3) with *E*_receptor_ being obtained from a theoceptor calculation in which the ligand had been deleted. The values of the coefficients were *α* = –0.080, *β* = 0.892 and *γ* = 0.419 and yield the relationship shown in Fig. 3. The value of *β*, when compared to those in Table 1, is consistent with a binding site that is hydrophobic in parts with a polar section, as included in the theoceptor. Protein–ligand crystal structures are interpretations that are several steps removed from the experimental measurements, and so include uncertainties, many of which depend on the force field used during refinement.^12^ QM calculations can help discriminate amongst plausible interpretations of the observed electron density. The chemical rigour demanded by theoceptor calculations can improve: ligand conformations, interpretations of the positioning of heteroatoms, stereoselectivity, positioning of ligands where electron density is missing, assignment of tautomeric and ionisation states.^87^ Further, by studying a set of conformations (and tautomers when relevant) of the free and bound ligand, differences between the low energy conformations and the bioactive conformation are identified and the conformer-focusing problem can be addressed.^88^ Compounds where one or other of these effects were relevant are described.

### 2.1 Compound **1**

**Fig. 4:**
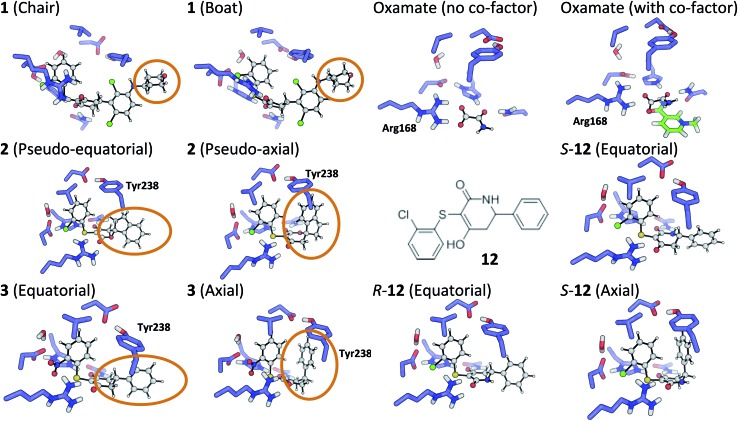
Theoceptor optimized structures described in the text.

The tetrahydropyran ring of ligand **1** is crystallographically modeled in a boat conformation that yields a predicted affinity far from experiment. This is corrected when modeled as a chair; the free conformation is 9 kcal mol^–1^ lower than the boat and the theoceptor with **1** in a chair conformation is 10 kcal mol^–1^ lower in energy than with a boat conformation (Fig. 4). The RSCC of the ligand was 0.94, which implies that the ligand fits the density well. However, the density contoured at 1*σ* reveals that both chair and boat would fit equally well; the differentiating part of the density is absent.

**Fig. 5:**
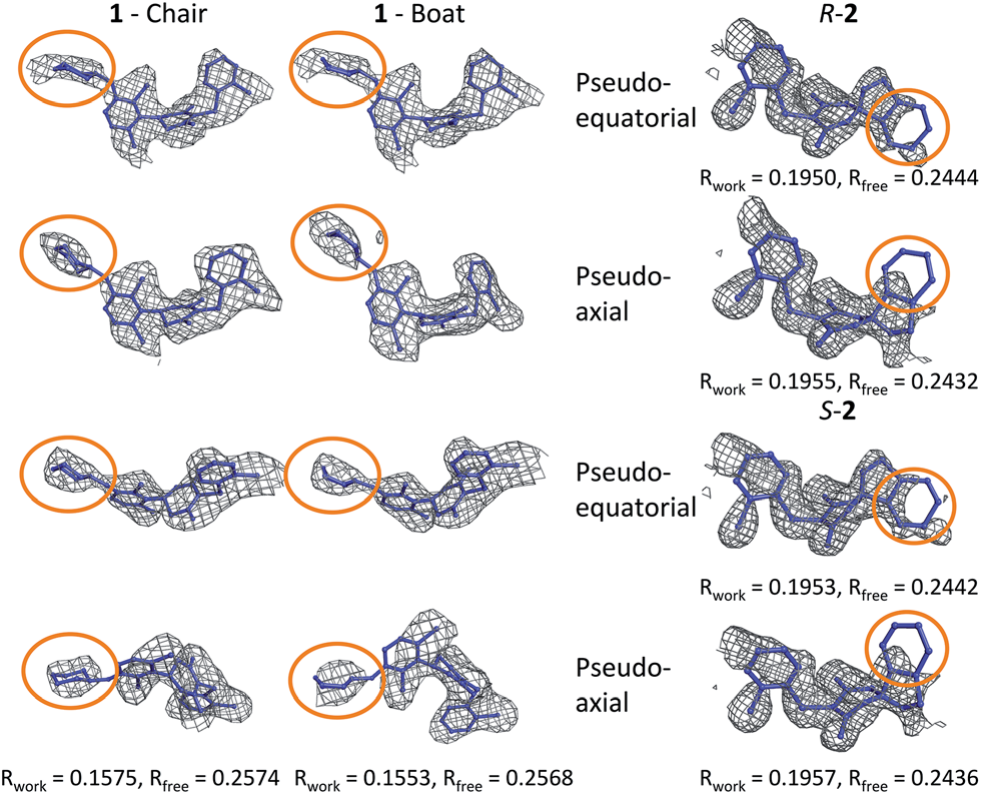
Re-refined structures and omit maps (see text).

In order to investigate this further, the deposited structure was re-refined (using the PHENIX suite of software)^41^,^88^ using two alternative sets of restraints on the conformation of the tetrahydropyran ring: one in which the ring is kept as a chair and one as a boat. In this structure, the ligand is present in all four protein molecules in the asymmetric unit. Subsequent to the single round of refinement, omit maps were generated in which the remainder of the structure is used to compute the phasing and the density in the omitted region (the ligand). These maps are shown for the four ligands present in the asymmetric unit in Fig. 5 and confirm that the available density cannot provide a definitive conformation for this ring. Better statistics were obtained for the boat conformation (the all boat structure achieved *R*_work_ of 0.1553 and *R*_free_ of 0.2568 while the all chair structure achieved *R*_work_ of 0.1575 and *R*_free_ of 0.2574). Inappropriate restraints and the statistics used to analyze the outcome misled the original refinement resulting in the wrong ligand conformation. Occasionally (see below) the correct conformation of a ring will not be so trivial and in these circumstances a quantum mechanical binding energy that accounts for the conformational preference of the ligand in the field of the protein can provide a very useful companion to a structural refinement.

### 2.2 Compound **2**

The RSCC for ligand **2** of 0.83 suggests that it does not fit the density map accurately and inspection reveals that the density around the indane moiety is missing. The first step to model this compound was to determine the preferred position of the oxygen atom in the dihydropyrone ring, because this can easily be misplaced when interpreting electron density maps.^12^ Changing the position of the oxygen atom inverts the configuration of the stereocentre. This compound was obtained as a single, unknown enantiomer and its absolute stereochemistry was assigned as *R* by Fauber *et al.* on the basis of the protein–ligand crystal structure.^68^ The energy difference between the theoceptor optimized with the oxygen in each of the two possible positions was less than 0.2 kcal mol^–1^, indicating that the ring oxygen is not interacting with the theoceptor and the proposed positioning is reasonable.^71^ The missing electron density around the indane moiety would be consistent with the phenyl substituent adopting pseudo-axial or pseudo-equatorial orientations, relative to the dihydropyrone ring. In the free ligand, the difference between these two conformations is less than 0.1 kcal mol^–1^ but in the complex with LDHA, the conformation with the phenyl in the pseudo-axial conformation is 8 kcal mol^–1^ lower in energy than in the pseudo-equatorial conformation (Fig. 4). This energy change originates from additional edge to face stacking between the ligand in its pseudo-axial conformation and tyrosine (Fig. 4, with the *R* stereochemistry retained for ease of comparison). We therefore propose a bound structure in which the position of the ring oxygen is maintained but the phenyl group is in the pseudo-axial position. This provides a better interpretation of the experimental data and suggests that the absolute stereochemistry is likely to have been misassigned based on the deposited crystal structure and is actually *S*.

To investigate this possibility, the deposited structure was re-refined. In this case, the ligand is only present in one of the proteins in the asymmetric unit and geometries of the two stereoisomers and two conformations were created in GaussView and were overlaid onto the ligand in the deposited structure using the pair_fit command in pymol.^89^,^90^ These initial positions were used to initiate a first round of refinement in PHENIX. The resulting ligand geometries were extracted and used to create a set of restraints in the eLBOW tool.^91^ These were then used during a second round of refinement in PHENIX that was analyzed with omit maps. The values of *R*_work_ and *R*_free_ obtained during this phase are provided in Fig. 5. The refinement statistics for the axial conformations are inferior to those for the equatorial. However, the maps in Fig. 5 reveal that the aromatic ring is not strongly supported in either position by the available experimental evidence. In this case, even though the structure has a reasonable overall resolution of 1.91 Å, expanding the evidence base for the structure to include theoceptor energies would permit a better-informed determination of the conformation and therefore the identification of the stereochemistry would be placed on a stronger footing. Ensuring that chemical insight (using knowledge or quantum mechanically derived energies) is involved in defining the conformational preferences of ligands when in protein binding sites should ensure that correct deductions are more likely to be made from these structures.

### 2.3 Compound **11**

As with the oxygen atom in **2**, the pyridine nitrogen in **11** is impossible to place with certainty at 2.2 Å resolution.^12^ In this case, the theoceptor finds an energy difference of 4 kcal mol^–1^ favouring the ligand with nitrogen oriented as in the deposited structure. This preference is due to an interaction of the pyridine nitrogen with a positively charged environment formed by the sidechains of His192 and Asn137. The theoceptor provides extra support for the deposited structure.

### 2.4 Compound **3**

The phenyl substituent in ligand **3** is in an axial position relative to the 3-hydroxycyclohexenone ring in the deposited structure. Somewhat surprisingly, axial and equatorial ligand conformations were computed to be energetically indistinguishable in the unbound state (energy difference = 0.6 kcal mol^–1^). In **3** there are no axial hydrogens located on the same face of the ring as the phenyl group and consequently, no clashes. However, there is a clear preference in the bound state: the theoceptor with the ligand in an equatorial conformation is 4 kcal mol^–1^ higher in energy. This difference originates from protein–ligand edge to face stacking present when the ligand is in an axial conformation (Fig. 5). In this case the theoceptor confirms the surprising conformation that had been proposed for **3**.

### 2.5 Compounds **8–10**

Initial positioning of the malonate derivatives was carried out by superimposing the malonate moiety on the 3-hydroxy cyclohexenone ring of ligand **3**, to place it in close proximity to the basic side chain of Arg168. The malonate moiety of compound **9** was first modeled in its dianionic form; the resulting complexation energy (Δ*E*) of +44 kcal mol^–1^ is not in the range found for other ligands described here. This arises due to the loss of solvation energy that is not compensated by sufficient interactions in the receptor. These findings suggest that the malonate moiety may bind in a mono-deprotonated form. Three different malonate positions were investigated for monoanionic **9** in the theoceptor. The lowest energy theoceptor was the one where the malonate moiety made four hydrogen bonds with the nearby sidechains (–18.9 kcal mol^–1^). The energy difference of 15 kcal mol^–1^ between the highest and lowest energy theoceptor emphasizes the effect subtle conformational changes of the interacting groups can have on the overall binding energy, particularly when strongly interacting groups such as anions are involved.

### 2.6 Malonate and oxamate

When considering the binding of malonate itself, a total of 8 different malonate tautomeric monoanions were optimized in solution and all were found to be energetically indistinguishable (within a range spanning 0.5 kcal mol^–1^). The correct orientation of malonate in the binding cavity could also not be confidently determined: optimization of 8 different theoceptors, each with a different malonate starting position resulted in complexes with Δ*E* values ranging from –8 to +16 kcal mol^–1^. These findings, in conjunction with experimental ambiguity concerning the state of the protein during the experimental measurements, suggested that malonate may bind in the presence of and proximal to cofactor NAD+/NADH.^74^ Calculations employed the oxidized and reduced form of nicotinamide with the ribose present but with the phosphate moiety truncated to a methyl group.^92^ The Δ*E* with co-factor in its reduced form was 5.4 kcal mol^–1^ and in its oxidized form almost –40 kcal mol^–1^, which is consistent with the experimental affinities. Finally, the binding of oxamate was investigated. Again, binding is facilitated by the co-factor: the energy difference between the theoceptor with and without co-factor bound was more than 50 kcal mol^–1^ (Fig. 4). These findings are very interesting from the drug designer's point of view: small negatively charged molecules that mimic substrate and bind in the presence of co-factor (rather than competing with it) have the tendency to be more efficient binders. As these studies were underway, a publication disclosed a new inhibitor, **12**, in which a nitrogen has been introduced into the central ring of compounds like **1**, **3**, **4** and **5**.^64^ This provided an opportunity to test the predictive capability of the theoceptor. The calculations for compound **12** yielded a Δ*E* of –19.5 kcal mol^–1^ and log *P* of 3.84. These gave a predicted pIC_50_ of 5.41. The experimental work had measured the pIC_50_ to be 5.40, which is in excellent agreement with the prediction. Furthermore, the calculations reveal that when the ligand is outside the protein, the pseudo-axial and pseudo-equatorial conformations are within 0.4 kcal mol^–1^ but in the binding site, the axial is preferred by 5 kcal mol^–1^. As with compounds **2** and **3**, this is due to edge to face interactions with tyrosine (Fig. 4). The favored structure sees the introduced NH group on the opposite side of the ligand to the NH in the sidechain of Asn137; the alternative position is computed to be 1 kcal mol^–1^ higher in energy. The preferred stereoisomer is therefore *S*-**12** (the original publication does not explore the stereochemical preference for this molecule).

## 3 Conclusions

The combination of computed quantum mechanical binding energies with measured or predicted ligand log *P* values can provide usefully accurate predictions of host–guest and protein–ligand binding energies. By assigning relative energies to protein–ligand structures in which different conformations, tautomers, protonation states and stereoisomers are sampled, useful insights about the interactions between the protein and ligand are generated. This can include regions that are not observed in the experimental electronic density. Computed structures reveal the limitations of the statistics currently used during the refinement of X-ray crystal structures. Our approach is sufficiently accurate to make useful predictions about the affinity for new compounds. Chemists must become proactive creators of protein–ligand crystal structures rather than passive consumers; quantum mechanics provides a useful tool to do this in an objective fashion.

## Conflicts of interest

AGL is a former employee and shareholder of AstraZeneca and is a shareholder of Medchemica Limited.
